# Chronic Obstructive Pulmonary Disease and Smoking Status — United States, 2017

**DOI:** 10.15585/mmwr.mm6824a1

**Published:** 2019-06-21

**Authors:** Anne G. Wheaton, Yong Liu, Janet B. Croft, Brenna VanFrank, Thomas L. Croxton, Antonello Punturieri, Lisa Postow, Kurt J. Greenlund

**Affiliations:** ^1^Division of Population Health, National Center for Chronic Disease Prevention and Health Promotion, CDC; ^2^Office on Smoking and Health, National Center for Chronic Disease Prevention and Health Promotion, CDC; ^3^Division of Lung Diseases, National Heart, Lung, and Blood Institute, National Institutes of Health.

Cigarette smoking is the leading cause of chronic obstructive pulmonary disease (COPD) in the United States; however, an estimated one fourth of adults with COPD have never smoked (*1*). CDC analyzed state-specific Behavioral Risk Factor Surveillance System (BRFSS) data from 2017, which indicated that, overall among U.S. adults, 6.2% (age-adjusted) reported having been told by a health care professional that they had COPD. The age-adjusted prevalence of COPD was 15.2% among current cigarette smokers, 7.6% among former smokers, and 2.8% among adults who had never smoked. Higher prevalences of COPD were observed in southeastern and Appalachian states, regardless of smoking status of respondents. Whereas the strong positive correlation between state prevalence of COPD and state prevalence of current smoking was expected among current and former smokers, a similar relationship among adults who had never smoked suggests secondhand smoke exposure as a potential risk factor for COPD. Continued promotion of smoke-free environments might reduce COPD among both those who smoke and those who do not.

Data from 418,378 adult respondents to the 2017 BRFSS survey in the 50 states and the District of Columbia (DC) were analyzed. BRFSS is an annual state-based, random-digit–dialed cellular and landline telephone survey of the noninstitutionalized U.S. population aged ≥18 years and is conducted by state health departments in collaboration with CDC.* Response rates for BRFSS are calculated using standards set by the American Association for Public Opinion Research (AAPOR) Response Rate Formula #4.^†^ The response rate is the number of respondents who completed the survey as a proportion of all eligible and likely eligible persons. The median survey response rate for all states and DC in 2017 was 45.9% and ranged from 30.6% to 64.1%.^§^ COPD was defined by an affirmative response to the question “Has a doctor, nurse, or other health professional ever told you that you had chronic obstructive pulmonary disease or COPD, emphysema, or chronic bronchitis?” Persons were considered to have never smoked if they reported never smoking or smoked less than 100 cigarettes during their lifetime. Former smokers had smoked at least 100 cigarettes in their life, but were not current smokers. Current smokers had smoked at least 100 cigarettes and currently smoked some days or every day.

Age-specific and age-adjusted^¶^ percentages and 95% confidence intervals (CIs) of adults with diagnosed COPD for all respondents and by smoking status were calculated for groups defined by selected sociodemographic characteristics, health characteristics, and state. Comparisons were made between these groups using t-tests with statistical significance set at p<0.05. State-specific age-adjusted current smoking prevalence was compared with state-specific age-adjusted COPD prevalence using Pearson correlation for all respondents and groups defined by smoking status. All analyses were conducted using SAS-callable SUDAAN (version 11.0.1; RTI International) to account for the stratified, complex cluster sampling design of the survey.

Overall age-adjusted prevalence of COPD was 6.2% in 2017 and was higher among women, older adults, and American Indians/Alaska Natives. Prevalence was also higher among those with less education, those who lived in more rural counties, those with a history of asthma, those who were underweight or obese, those who reported no leisure-time physical activity in the past 30 days, and those with additional chronic conditions (Table 1). Similar patterns were observed irrespective of smoking status. Among all adults, age-adjusted prevalence of COPD ranged from 3.4% in Hawaii to 13.8% in West Virginia (Table 2) (Figure). Among current smokers, overall age-adjusted COPD prevalence was 15.2% and ranged from 7.8% in Hawaii to 25.9% in West Virginia. Among former smokers, age-adjusted COPD prevalence was 7.6% and ranged from 4.7% in Hawaii to 15.1% in West Virginia. Among adults who never smoked, age-adjusted COPD prevalence was 2.8% and ranged from 1.6% in Minnesota to 6.0% in West Virginia. Among current smokers, COPD prevalence was highest in states in the Southeast and the Midwest. Among adults who never smoked, states with the highest COPD prevalence were concentrated in the Southeast. State-level prevalence of COPD among current smokers was strongly correlated with state-level current smoking prevalence (Pearson correlation coefficient = 0.69, p<0.001). State-level COPD prevalence among former smokers (Pearson correlation coefficient = 0.71, p<0.001) and among adults who had never smoked (Pearson correlation coefficient = 0.64, p<0.001) also were strongly correlated with state-level current smoking prevalence.

**TABLE 1:** Age-specific and age-adjusted* percentage of adults aged ≥18 years with COPD, by smoking status and selected characteristics — Behavioral Risk Factor Surveillance System, 2017

Characteristic	All adults	Current smokers	Former smokers	Never smokers
	% (95% CI)	% (95% CI)	% (95% CI)	% (95% CI)
**Overall**	**6.2 (6.0–6.3)**	**15.2 (14.7–15.7)**	**7.6 (7.3–8.0)**	**2.8 (2.7–2.9)**
**Sex**				
Men	5.5 (5.4–5.7)	12.4 (11.8–13.1)	6.6 (6.2–7.1)	2.3 (2.1–2.5)
Women	6.8 (6.6–7.0)	18.5 (17.8–19.3)	8.9 (8.4–9.5)	3.2 (3.0–3.4)
**Age group (yrs)**				
18–44	2.7 (2.5–2.8)	6.8 (6.2–7.3)	2.9 (2.5–3.4)	1.4 (1.3–1.6)
45–54	6.3 (6.0–6.7)	17.7 (16.4–19.1)	7.1 (6.4–8.0)	2.5 (2.2–2.9)
55–64	10.6 (10.2–11.0)	25.8 (24.4–27.3)	12.6 (11.9–13.5)	4.1 (3.7–4.5)
≥65	12.8 (12.5–13.2)	30.1 (28.5–31.8)	17.5 (16.8–18.3)	6.1 (5.7–6.5)
**Race/Ethnicity**				
White^†^	6.7 (6.5–6.8)	16.9 (16.3–17.5)	7.8 (7.5–8.2)	2.7 (2.5–2.8)
Black^†^	6.6 (6.1–7.1)	11.2 (10.0–12.6)	8.8 (7.3–10.5)	4.1 (3.6–4.7)
Hispanic	3.6 (3.2–3.9)	8.0 (6.5–9.8)	5.2 (4.2–6.3)	2.3 (1.9–2.6)
American Indian/Alaska Native^†^	11.9 (10.3–13.7)	21.6 (18.0–25.8)	10.9 (8.5–14.0)	5.7 (4.1–7.8)
Asian^†^	1.7 (1.2–2.5)	8.3 (4.3–15.3)^§^	1.5 (0.9–2.6)	1.2 (0.7–2.1)
Native Hawaiian/Pacific Islander^†^	3.3 (1.8–6.0)^§^	14.9 (7.3–27.9)^§^	4.3 (2.2–8.2)^§^	0.9 (0.4–2.1)^§^
Other/Multiracial^†^	9.3 (8.3–10.5)	19.4 (16.9–22.2)	10.3 (8.8–12.1)	3.9 (2.8–5.6)
**Education level**				
Less than high school diploma	10.4 (9.9–11.0)	20.0 (18.7–21.4)	12.5 (11.1–14.0)	4.0 (3.5–4.5)
High school diploma	7.4 (7.1–7.7)	14.9 (14.1–15.7)	8.8 (8.2–9.4)	3.5 (3.1–3.9)
Some college	6.5 (6.2–6.7)	14.6 (13.7–15.5)	7.3 (6.9–7.9)	3.3 (3.0–3.6)
College graduate	2.7 (2.6–2.9)	8.6 (7.8–9.6)	4.4 (3.9–4.9)	1.6 (1.4–1.7)
**Urban-rural status^¶^**				
Large metropolitan center	4.8 (4.5–5.1)	11.8 (10.8–12.9)	6.0 (5.4–6.7)	2.6 (2.3–2.9)
Large fringe metropolitan	5.7 (5.4–6.0)	14.7 (13.6–15.9)	7.1 (6.4–7.8)	2.6 (2.4–2.9)
Medium metropolitan	6.5 (6.3–6.8)	16.1 (15.1–17.1)	8.0 (7.3–8.6)	2.9 (2.6–3.1)
Small metropolitan	7.3 (7.0–7.7)	17.0 (15.8–18.3)	9.1 (8.2–10.0)	3.0 (2.7–3.3)
Micropolitan	8.3 (7.9–8.8)	18.2 (17.0–19.4)	10.2 (9.2–11.2)	3.2 (2.8–3.5)
Noncore	8.5 (8.0–9.0)	18.8 (17.3–20.3)	9.7 (8.7–10.8)	3.6 (3.2–4.1)
**Ever had asthma**				
Yes	19.5 (19.0–20.1)	37.3 (35.9–38.9)	21.3 (20.2–22.5)	11.2 (10.6–11.9)
No	4.1 (4.0–4.2)	10.9 (10.4–11.4)	5.3 (5.0–5.6)	1.6 (1.5–1.8)
**Body mass index (BMI, kg/m^2^)**				
Underweight (BMI<18.5)	13.6 (11.8–15.5)	25.7 (22.3–29.5)	18.8 (13.5–25.6)	3.4 (2.1–5.5)
Normal weight (BMI = 18.5–24.9)	5.7 (5.5–6.0)	14.9 (14.1–15.8)	6.9 (6.3–7.5)	2.1 (1.8–2.4)
Overweight (BMI = 25.0–29.9)	4.9 (4.7–5.1)	12.6 (11.8–13.4)	6.5 (5.9–7.2)	2.0 (1.9–2.2)
Obesity (BMI≥30.0)	8.1 (7.8–8.4)	17.9 (16.9–19.0)	9.4 (8.8–10.0)	4.5 (4.2–4.8)
Leisure-time physical activity**				
Yes	4.8 (4.7–5.0)	12.7 (12.1–13.3)	6.0 (5.7–6.3)	2.3 (2.2–2.5)
No	9.6 (9.3–9.9)	19.2 (18.3–20.2)	11.9 (11.0–12.9)	4.1 (3.8–4.4)
**Number of chronic conditions^††^**				
None	2.5 (2.4–2.7)	6.5 (5.8–7.1)	3.7 (3.3–4.1)	1.2 (1.1–1.3)
1	5.8 (5.5–6.1)	13.4 (12.5–14.2)	6.5 (5.9–7.0)	2.7 (2.5–3.0)
2	12.6 (11.9–13.4)	24.5 (22.9–26.2)	13.5 (11.1–16.3)	6.4 (5.5–7.4)
3	20.2 (18.1–22.5)	32.1 (28.5–36.0)	22.6 (17.4–28.8)	11.7 (9.1–15.0)
≥4	34.4 (30.3–38.8)	45.7 (39.9–51.6)	42.6 (37.5–47.8)	25.3 (19.3–32.3)

**Abbreviations:** CI = confidence interval; COPD = chronic obstructive pulmonary disease.

* Percentages for all characteristics except age group were age-adjusted to the 2000 U.S. standard population aged ≥18 years.

^†^ Non-Hispanic.

^§^ Unreliable estimate because relative standard error >0.3.

^¶^ Classification based on the National Center for Health Statistics (NCHS) 2013 Urban-Rural Classification Scheme for Counties, which uses 2010 U.S. Census population data and the February 2013 Office of Management and Budget designations of metropolitan statistical area, micropolitan statistical area, or noncore area. https://www.cdc.gov/nchs/data/series/sr_02/sr02_166.pdf.

** Any leisure-time physical activity in the past 30 days.

^††^ Chronic conditions include coronary heart disease (heart attack, angina, or coronary heart disease), stroke, diabetes, cancer, arthritis, kidney disease, and depressive disorder.

**TABLE 2:** Age-adjusted* percentage of adults aged ≥18 years with diagnosed COPD, by smoking status and state — Behavioral Risk Factor Surveillance System, 2017

State	Total (N = 418,378)	Current smokers (n = 61,855)		Former smokers (n = 118,692)		Never smoked (n = 237,831)	
	% with COPD (95% CI)	% of total (95% CI)	% with COPD (95% CI)	% of total (95% CI)	% with COPD (95% CI)	% of total (95% CI)	% with COPD (95% CI)
**Total**	**6.2 (6.0–6.3)**	**16.9 (16.6–17.1)**	**15.2 (14.7–15.7)**	**23.0 (22.8–23.3)**	**7.6 (7.3–8.0)**	**60.1 (59.8–60.4)**	**2.8 (2.7–2.9)**
Alabama	10.1 (9.2–11.2)	22.0 (20.5–23.6)	22.7 (19.7–25.9)	22.1 (20.8–23.5)	12.2 (10.2–14.5)	55.9 (54.2–57.6)	4.3 (3.6–5.1)
Alaska	6.3 (4.8–8.2)	20.8 (18.2–23.6)	14.0 (9.5–20.1)	26.4 (24.2–28.8)	5.1 (3.6–7.2)	52.8 (49.9–55.7)	3.2 (1.9–5.2)
Arizona	5.9 (5.5–6.4)	15.9 (15.1–16.8)	13.9 (12.4–15.6)	23.3 (22.5–24.2)	7.9 (6.7–9.3)	60.8 (59.7–61.9)	2.6 (2.3–3.1)
Arkansas	9.3 (8.1–10.8)	23.4 (21.1–26.0)	21.4 (17.7–25.6)	24.3 (22.2–26.6)	12.0 (7.6–18.4)	52.2 (49.5–54.9)	3.6 (2.7–4.7)
California	4.4 (3.9–4.9)	11.6 (10.6–12.7)	11.0 (8.7–13.9)	21.7 (20.6–22.8)	6.7 (5.4–8.3)	66.7 (65.3–68.0)	2.2 (1.8–2.7)
Colorado	4.2 (3.7–4.7)	14.7 (13.7–15.7)	12.1 (10.2–14.3)	25.4 (24.3–26.5)	4.9 (4.1–5.9)	59.9 (58.7–61.2)	1.7 (1.3–2.1)
Connecticut	5.3 (4.7–5.9)	13.4 (12.3–14.6)	14.7 (12.2–17.7)	24.4 (23.3–25.5)	7.2 (5.6–9.3)	62.2 (60.7–63.6)	2.6 (2.0–3.3)
DC	5.8 (5.0–6.7)	14.8 (13.5–16.2)	15.5 (11.6–20.4)	19.5 (18.1–21.0)	6.1 (4.7–8.0)	65.7 (63.9–67.5)	2.9 (2.3–3.7)
Delaware	7.3 (6.2–8.5)	18.0 (16.2–20.0)	19.2 (15.4–23.6)	23.7 (21.8–25.8)	8.8 (6.7–11.3)	58.2 (55.9–60.5)	2.5 (1.7–3.5)
Florida	7.1 (6.3–8.0)	16.8 (15.5–18.1)	15.7 (13.5–18.2)	22.4 (21.2–23.7)	8.2 (6.6–10.3)	60.8 (59.1–62.4)	3.9 (2.9–5.1)
Georgia	6.8 (6.1–7.6)	17.8 (16.4–19.2)	16.4 (13.6–19.6)	20.0 (18.8–21.3)	9.4 (6.8–12.9)	62.2 (60.6–63.9)	3.4 (2.8–4.1)
Hawaii	3.4 (3.0–3.9)	13.5 (12.4–14.8)	7.8 (5.8–10.5)	25.8 (24.3–27.2)	4.7 (3.7–5.9)	60.7 (59.1–62.3)	1.9 (1.5–2.4)
Idaho	4.7 (4.1–5.5)	14.8 (13.4–16.4)	13.1 (10.3–16.5)	23.0 (21.4–24.6)	5.6 (4.3–7.3)	62.2 (60.2–64.2)	2.1 (1.5–2.8)
Illinois	6.4 (5.7–7.3)	15.7 (14.4–17.2)	15.2 (12.5–18.4)	22.5 (21.1–23.9)	7.7 (6.1–9.7)	61.8 (60.1–63.5)	2.9 (2.3–3.7)
Indiana	8.0 (7.5–8.6)	22.5 (21.5–23.6)	18.3 (16.6–20.1)	23.9 (22.9–24.9)	8.5 (7.5–9.5)	53.6 (52.4–54.8)	3.3 (2.8–3.9)
Iowa	5.9 (5.3–6.5)	17.9 (16.8–19.1)	16.4 (14.1–19.0)	24.0 (22.9–25.2)	8.1 (5.8–11.3)	58.0 (56.7–59.4)	2.2 (1.8–2.8)
Kansas	6.2 (5.8–6.6)	18.0 (17.3–18.8)	16.3 (14.9–17.8)	23.8 (23.0–24.5)	7.9 (7.1–8.7)	58.2 (57.3–59.1)	2.4 (2.1–2.7)
Kentucky	11.3 (10.2–12.5)	25.5 (23.9–27.2)	23.7 (20.7–26.9)	24.6 (23.0–26.1)	11.3 (9.4–13.5)	49.9 (48.2–51.7)	4.3 (3.4–5.4)
Louisiana	8.4 (7.4–9.5)	23.8 (22.1–25.6)	16.4 (13.5–19.7)	22.0 (20.5–23.5)	11.2 (9.0–13.9)	54.2 (52.3–56.1)	3.5 (2.8–4.4)
Maine	6.5 (5.8–7.3)	18.7 (17.2–20.3)	16.4 (14.1–18.9)	29.0 (27.5–30.5)	8.9 (6.7–11.7)	52.3 (50.6–54.1)	1.9 (1.4–2.5)
Maryland	5.4 (4.8–6.0)	14.1 (13.1–15.2)	14.0 (11.7–16.7)	20.9 (19.9–21.9)	6.3 (5.3–7.5)	65.0 (63.7–66.3)	2.7 (2.2–3.3)
Massachusetts	5.0 (4.3–5.8)	14.1 (12.7–15.6)	15.2 (11.8–19.3)	23.5 (22.0–25.2)	5.7 (4.5–7.2)	62.4 (60.4–64.2)	1.8 (1.3–2.5)
Michigan	8.0 (7.3–8.6)	20.4 (19.3–21.5)	18.6 (16.5–20.9)	25.3 (24.2–26.4)	8.4 (7.3–9.6)	54.3 (53.0–55.6)	3.3 (2.8–4.0)
Minnesota	4.0 (3.7–4.4)	14.7 (14.0–15.5)	10.5 (9.1–12.1)	25.6 (24.7–26.4)	5.1 (4.4–5.9)	59.7 (58.7–60.7)	1.6 (1.3–2.0)
Mississippi	7.5 (6.6–8.5)	22.9 (21.0–24.9)	15.4 (12.5–18.8)	20.7 (19.1–22.3)	8.9 (6.9–11.3)	56.4 (54.3–58.6)	3.3 (2.5–4.2)
Missouri	7.9 (7.1–8.6)	21.6 (20.2–23.2)	19.1 (16.6–21.8)	24.8 (23.4–26.3)	8.6 (7.3–10.0)	53.6 (51.8–55.3)	3.1 (2.5–3.7)
Montana	5.7 (4.9–6.5)	18.4 (16.9–20.0)	12.9 (10.3–15.9)	25.8 (24.2–27.5)	7.1 (5.8–8.6)	55.8 (53.9–57.7)	2.3 (1.7–3.0)
Nebraska	5.3 (4.8–5.8)	15.9 (14.9–16.9)	14.6 (12.6–16.9)	24.0 (22.9–25.1)	6.4 (5.4–7.5)	60.1 (58.8–61.4)	2.2 (1.8–2.7)
Nevada	6.5 (5.5–7.6)	17.5 (15.6–19.6)	14.4 (10.9–18.8)	22.7 (20.8–24.8)	7.9 (5.9–10.5)	59.8 (57.4–62.2)	3.2 (2.3–4.4)
New Hampshire	6.0 (5.2–7.0)	17.0 (15.2–19.0)	16.4 (13.0–20.6)	28.3 (26.5–30.1)	7.2 (5.7–9.1)	54.7 (52.5–56.9)	2.5 (1.9–3.3)
New Jersey	5.8 (5.1–6.5)	14.1 (12.9–15.4)	12.8 (10.6–15.3)	23.9 (22.6–25.3)	6.3 (5.2–7.7)	62.0 (60.4–63.6)	3.6 (2.8–4.6)
New Mexico	5.6 (4.9–6.4)	17.9 (16.4–19.4)	13.2 (10.7–16.3)	22.9 (21.4–24.4)	7.0 (5.5–9.0)	59.3 (57.4–61.0)	2.5 (1.9–3.2)
New York	5.0 (4.5–5.5)	14.4 (13.5–15.4)	11.9 (10.2–13.8)	22.1 (21.1–23.2)	5.8 (5.0–6.8)	63.5 (62.2–64.7)	2.8 (2.3–3.3)
North Carolina	7.3 (6.4–8.2)	17.5 (16.0–19.1)	16.4 (13.4–20.0)	24.9 (23.3–26.5)	7.7 (6.3–9.4)	57.7 (55.8–59.6)	3.5 (2.8–4.5)
North Dakota	4.8 (4.2–5.4)	18.9 (17.6–20.3)	12.5 (10.4–15.1)	25.2 (23.9–26.6)	4.8 (3.9–5.9)	55.8 (54.2–57.5)	1.8 (1.4–2.4)
Ohio	7.6 (6.9–8.2)	22.1 (20.8–23.4)	16.7 (14.7–18.8)	23.4 (22.2–24.5)	9.5 (8.1–11.1)	54.6 (53.1–56.0)	2.9 (2.4–3.6)
Oklahoma	8.1 (7.3–8.9)	20.5 (19.1–22.0)	17.7 (15.3–20.4)	23.8 (22.5–25.2)	10.6 (9.0–12.5)	55.7 (54.0–57.4)	3.2 (2.6–3.9)
Oregon	4.9 (4.3–5.6)	16.7 (15.4–18.1)	12.6 (10.3–15.5)	24.5 (23.2–25.9)	6.2 (4.7–8.0)	58.8 (57.1–60.4)	2.0 (1.5–2.6)
Pennsylvania	5.9 (5.3–6.7)	19.7 (18.3–21.1)	11.6 (9.5–14.0)	25.6 (24.2–27.0)	8.8 (7.2–10.7)	54.8 (53.1–56.5)	2.2 (1.7–2.9)
Rhode Island	7.0 (6.1–8.1)	15.5 (13.9–17.3)	16.2 (13.0–20.0)	26.7 (25.0–28.4)	10.2 (7.8–13.2)	57.8 (55.7–59.8)	2.5 (1.9–3.4)
South Carolina	7.2 (6.6–7.9)	19.7 (18.5–20.9)	16.9 (14.9–19.2)	25.2 (24.1–26.4)	8.0 (6.5–9.8)	55.1 (53.7–56.5)	3.5 (2.9–4.2)
South Dakota	4.4 (3.6–5.4)	20.6 (18.5–22.8)	10.2 (7.3–14.2)	25.0 (23.0–27.1)	5.0 (3.8–6.7)	54.5 (52.1–56.8)	2.0 (1.3–2.9)
Tennessee	8.9 (8.0–9.8)	23.3 (21.6–25.1)	19.7 (17.2–22.5)	22.8 (21.3–24.3)	9.9 (8.2–11.9)	54.0 (52.0–55.9)	3.7 (2.8–4.8)
Texas	4.8 (4.1–5.7)	16.0 (14.5–17.5)	13.3 (10.3–17.1)	19.9 (18.5–21.4)	6.3 (4.7–8.4)	64.1 (62.2–65.9)	2.4 (1.8–3.2)
Utah	4.1 (3.6–4.6)	9.0 (8.3–9.8)	12.3 (9.9–15.3)	15.6 (14.7–16.5)	6.1 (5.0–7.4)	75.4 (74.3–76.4)	2.4 (2.0–2.9)
Vermont	5.7 (5.1–6.4)	17.3 (15.8–18.9)	17.3 (14.6–20.4)	27.5 (26.0–29.1)	6.2 (5.1–7.5)	55.2 (53.3–57.0)	1.9 (1.5–2.4)
Virginia	6.6 (5.9–7.4)	16.8 (15.7–18.0)	16.2 (13.8–19.0)	23.1 (21.9–24.3)	9.1 (6.8–11.9)	60.1 (58.7–61.6)	2.9 (2.4–3.6)
Washington	5.4 (5.0–6.0)	13.8 (13.0–14.7)	15.5 (13.3–17.9)	26.3 (25.4–27.3)	7.1 (6.0–8.3)	59.8 (58.7–61.0)	2.0 (1.7–2.4)
West Virginia	13.8 (12.7–15.0)	28.1 (26.4–29.9)	25.9 (23.3–28.8)	24.4 (22.9–25.9)	15.1 (12.6–18.0)	47.5 (45.7–49.4)	6.0 (5.0–7.3)
Wisconsin	4.7 (4.0–5.5)	16.7 (15.2–18.2)	14.0 (11.1–17.4)	25.0 (23.4–26.6)	4.9 (3.9–6.2)	58.4 (56.5–60.2)	1.9 (1.4–2.6)
Wyoming	6.1 (5.3–6.9)	19.2 (17.6–21.0)	12.9 (10.3–16.1)	25.1 (23.5–26.8)	8.7 (7.1–10.6)	55.7 (53.7–57.7)	2.3 (1.8–3.0)

**Abbreviations:** CI = confidence interval; COPD = chronic obstructive pulmonary disease; DC = District of Columbia.

* Age-adjusted to the 2000 U.S. standard population aged ≥18 years.

**FIGURE:**
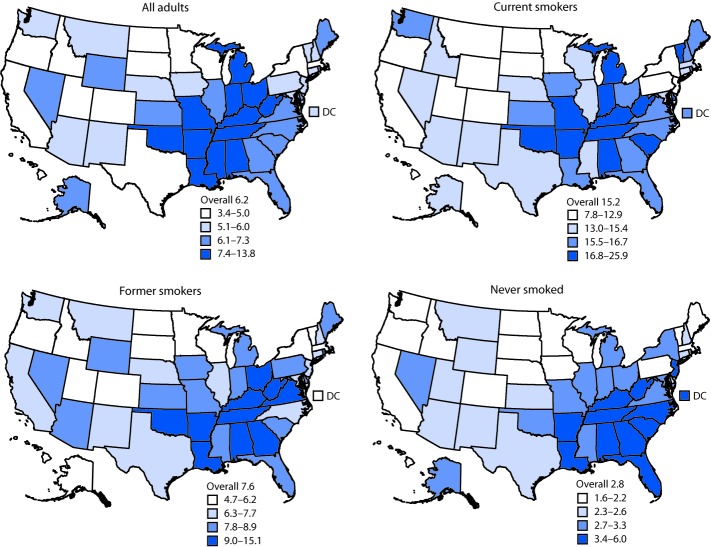
Age-adjusted* percentage of U.S. adults with chronic obstructive pulmonary disease (COPD), overall and by current or previous smoking status — Behavioral Risk Factor Surveillance System, 2017 **Abbreviation:** DC = District of Columbia. * Age-adjusted to the 2000 U.S. standard population aged ≥18 years.

## Discussion

The higher COPD prevalences observed among women, older adults, American Indians/Alaska Natives, adults with less education, those with a history of asthma, and those residing in rural areas were consistent with results from previous studies (*1*–*3*). The geographic distribution also was consistent (*1*). These patterns were similar among adults who had never smoked. Although smoking tobacco is the main contributor to COPD in the United States, other factors might play a role in the development of COPD among nonsmokers, including secondhand smoke exposure, occupational and environmental exposures, and chronic asthma (*4*,*5*). Secondhand smoke exposure, in either childhood or as an adult, has been associated with an increased risk for COPD-associated mortality (*6*). The 2006 Surgeon General’s report on secondhand smoke concluded that although the evidence suggested a causal relationship between exposure to secondhand smoke and COPD risk, there was insufficient evidence to state definitively that the relationship is causal (*7*).

In the current analysis, the geographic distribution of high COPD prevalence was similar for current smokers and adults who never smoked. There is also a strong correlation between state-level prevalences of COPD among adults who never smoked and state-level prevalence of current smoking. This could reflect that in certain regions adults who never smoked might be more likely to be exposed to secondhand smoke. Among the states in the highest quartile for COPD among adults who never smoked, only New Jersey had laws banning smoking in private worksites, restaurants, and bars as of December 31, 2017; the remainder of states in that quartile either had no smoke-free laws or laws banning smoking in only one or two venues.**

The findings in this report are subject to at least seven limitations. First, COPD status was based on self-report, not on medical records or diagnostic tests, and might be subject to recall and social desirability biases. Second, physicians might be more likely to diagnose COPD and other smoking-related diseases in states with high smoking rates, whereas COPD might be more likely to remain undiagnosed in states with lower smoking rates. Third, smoking status also was based on self-report and might be subject to social desirability bias. Fourth, because the data were cross-sectional, causality could not be examined. Fifth, e-cigarette use was not examined in this report. There were no other measures of exposure to secondhand smoke or other indoor or outdoor air pollutants or history of respiratory infections, all of which might contribute to COPD risk. Sixth, BRFSS surveys noninstitutionalized adults and does not include adults who live in long-term care facilities, prisons, and other facilities; therefore, findings are not generalizable to those populations. Finally, state BRFSS response rates were relatively low, which might lead to selection bias.

Population-based strategies for smoking prevention and control have the potential to decrease the prevalence of COPD in the United States. Such strategies include tobacco product price increases, mass media antismoking campaigns, comprehensive smoke-free laws, and barrier-free access to evidence-based cessation interventions.^††^ Comprehensive smoke-free laws not only help protect nonsmokers from secondhand smoke exposure, but they can also promote adoption of voluntary smoke-free rules in private settings (e.g., homes and automobiles) and reduce smoking prevalence through increased cessation and decreased initiation.^§§^ Clinicians can play a key role in increasing access to and use of cessation therapies, including counseling and Food and Drug Administration-approved cessation medications.^¶¶^ Current clinical guidelines recommend screening all patients for tobacco use at every visit (*8*); however, clinicians should be mindful that not all COPD is necessarily caused by smoking and should use spirometry for diagnosis in patients with COPD symptoms (*9*), regardless of their smoking history.

**Summary**

**What is already known about this topic?**

Cigarette smoking is the primary risk factor for chronic obstructive pulmonary disease (COPD) in the United States; an estimated one fourth of adults with COPD have never smoked. Higher COPD prevalence has been observed in southeastern and Appalachian states.

**What is added by this report?**

Geographic and sociodemographic patterns of COPD prevalence were similar among current smokers, former smokers, and adults who had never smoked.

**What are the implications for public health practice?**

Population-based strategies for smoking prevention and control, including comprehensive smoke-free policies, have the potential to decrease COPD prevalence, including among nonsmokers. Clinicians should offer cessation support to patients who smoke and consider COPD in symptomatic patients, regardless of smoking history.

## Footnotes

*
https://www.cdc.gov/brfss/about/index.htm .

†
http://www.aapor.org/AAPOR_Main/media/publications/Standard-Definitions20169theditionfinal.pdf .

§
https://www.cdc.gov/brfss/annual_data/2017/pdf/2017-sdqr-508.pdf .

¶ Age-adjusted to the 2000 U.S. standard population aged ≥18 years.

††
https://www.cdc.gov/tobacco/stateandcommunity/best_practices/index.htm .

§§
https://www.cdc.gov/tobacco/basic_information/secondhand_smoke/ .

¶¶
https://www.uspreventiveservicestaskforce.org/Page/Document/UpdateSummaryFinal/tobacco-use-in-adults-and-pregnant-women-counseling-and-interventions1 .
